# Access to essential medicines for mothers and children: regulatory challenges and gender justice

**DOI:** 10.1080/20523211.2026.2683095

**Published:** 2026-06-17

**Authors:** Aga Natalis, Abdul Jalil

**Affiliations:** Faculty of Law, Universitas Diponegoro, Semarang City, Indonesia

Dear Editor-in-Chief,

We are particularly interested in the findings of the study conducted by Briggs et al. (2026) regarding the registration status of medicines for maternal, newborn, and child health (MNCH) across nine countries. The study revealed that national registration processes continue to face significant structural, procedural, and legal barriers. On average, only 72% of the MNCH tracer medicines had at least one registered product, with figures ranging from 28% to 100%. This highlights unequal access to essential medicines, particularly in countries with limited regulatory capacity such as Nepal, Mali, and Senegal. Identified barriers included complex procedures, lengthy registration timelines, reliance on approvals from other regulatory authorities, and the absence of legal mechanisms to prioritise essential MNCH medicines.

In the context of Indonesia, these findings are highly relevant. Indonesia, as a developing country with a regulatory system currently being strengthened through the National Agency of Drug and Food Control (BPOM), faces similar challenges in ensuring the availability of essential medicines (Puspitasari et al., 2025). As the study explains, delays in registration can pose serious health risks, especially for pregnant women and newborns who require medicines such as oxytocin, misoprostol, and magnesium sulphate to manage postpartum haemorrhage and pre-eclampsia. This underscores the importance of integrating clear legal mechanisms within pharmaceutical regulations to ensure priority access to medicines critical for public health. Such measures align with Indonesian health law principles, including Law No. 17 of 2023 and BPOM regulations, which emphasise safety, quality, and the availability of medicines to the population.

From the perspective of feminist legal theory, this study can be analysed through the lens of gendered disparities in access to healthcare. According to MacKinnon (2007), legal structures and regulations often systematically reproduce gender inequalities, which can be observed in the context of reproductive health and access to critical medicines for women. Inadequate regulation or slow registration processes not only hinder access to essential medicines but also indirectly reinforce the marginalisation of women, who are the most vulnerable to maternal mortality and neonatal morbidity.

Moreover, the study highlights the importance of regulatory harmonisation, recognition of WHO prequalification, and the use of electronic information systems to accelerate registration processes. In Indonesia, this could be implemented through the digitalisation of BPOM’s registration system, including the use of reliance mechanisms based on decisions by recognised international authorities to expedite approval of essential MNCH medicines. By adopting a risk-based approach, prioritising critical medicines, and strengthening regulatory human resources, Indonesia can improve access to essential medicines for mothers and children more efficiently.

The research also indicates that local production of medicines does not necessarily correlate with higher registration rates, emphasising the need for an integrated government strategy that links domestic pharmaceutical development with regulatory reform. In the national legal context, this requires a clear juridical foundation that allows formal recognition of WHO pre-qualified products, cross-border recognition, and legally codified priority registration mechanisms. This approach not only enhances the efficiency of the regulatory system but also affirms the state’s commitment to fulfilling the right to health, particularly for women and children, as guaranteed by the constitution and ratified international human rights instruments.

Furthermore, the study underscores the critical role of market transparency and reducing marginal costs for pharmaceutical manufacturers in the registration process. This is consistent with feminist legal theory, which highlights how structural regulatory barriers disproportionately affect women and children. By reducing unnecessary administrative obstacles and costs, the state can ensure equitable availability of critical medicines, reduce gender disparities in healthcare access, and strengthen women’s position within the national health system.

Consequently, the findings of Briggs et al. (2026) demonstrate that evidence-based, legally grounded, and gender-sensitive regulatory reform is crucial for improving the availability of MNCH medicines. In Indonesia, implementing these recommendations could involve revising laws and BPOM regulations to strengthen priority registration mechanisms and reliance on WHO or other trusted authority decisions, digitalising and increasing transparency in registration systems, enhancing the capacity of regulatory personnel, and integrating domestic medicine development with national health policy. Through these measures, Indonesia can reduce maternal and neonatal mortality, improve the quality of healthcare services, and achieve social and gender justice in access to essential medicines.

This approach aligns with the philosophy of feminist legal theory, which emphasises that legal structures and regulations should proactively address inequalities, particularly in the context of women’s reproductive health. When medicine registration policies account for accessibility, safety, and quality while prioritising the most vulnerable groups, such as mothers and children, the state not only fulfils its legal and public health obligations but also implements substantive gender justice within pharmaceutical regulatory practice (Mike, 2020). Gender-responsive regulatory reform can serve as a model for other developing countries facing similar challenges in ensuring the availability of essential MNCH medicines (Lingam et al., 2025; Morgan et al., 2024).

Therefore, this study makes an important contribution to the development of pharmaceutical policy in Indonesia, offering empirical guidance for legislators, regulators, and stakeholders to enforce safe, effective, and gender-equitable access to MNCH medicines. The recommended legal, administrative, and technical measures can improve regulatory effectiveness, reduce production and distribution obstacles, and safeguard the rights of women and children to adequate healthcare, consistent with Indonesia’s vision of equitable health law and policy.
